# Neuroendocrine tumor of the appendix masquerading as acute appendicitis with a mucocele on CT scan: a rare finding

**DOI:** 10.1186/s40792-024-01870-5

**Published:** 2024-03-19

**Authors:** Abdullah S. Al-Darwish, Waad Rashaid Alsubaie, Waleed AlShammari, Muath AlaSheikh, Muath AlRashed

**Affiliations:** 1 Department of Surgery, Prince Mohammed Bin Abdulaziz Hospital, Riyadh, Saudi Arabia; 2 Department of Surgery, Arryan Hospital, Dr. Sulaiman Al Habib Medical Group, Riyadh, Saudi Arabia; 3 Department of Pathology, Prince Mohammed Bin Abdulaziz Hospital, Riyadh, Saudi Arabia; 4 Department of Surgery, King Saud University Medical City, Riyadh, Saudi Arabia

**Keywords:** Mucocele, Appendix, Neuroendocrine tumor, Laparoscopy, Appendectomy, Right hemicolectomy

## Abstract

**Introduction:**

Neuroendocrine tumors (NETs) of the appendix are rare and are often discovered incidentally during surgery for acute appendicitis or other unrelated conditions (Modlin et al. in Gastroenterology 128:1717–1751, 2005, Alsaad et al. in Oncol Rep 16:1105–1109, 2006, Frilling et al. in Lancet Oncol 15:e8–e21, 2014). These tumors can range from asymptomatic incidental findings to clinically significant tumors with metastases (Alsaad et al. in Oncol Rep 16:1105–1109, 2006, Gomes et al. in World J Emerg Surg 10:60, 2015, Paiva et al. in Eur J Cancer 38:702–705, 2002, Burke et al. in Am J Surg Pathol. 9:661–674, 1985). This case report presents a rare case of a NET of the appendix presenting as acute appendicitis.

**Case description:**

A 23-year-old male presented with right lower quadrant abdominal pain, nausea, and vomiting for 2 days. A CT scan revealed a mucocoele of the appendix. The patient underwent laparoscopic appendectomy, and the appendix was sent for histopathological examination. The final pathological report confirmed a NET of the appendix with a Ki-67 index of 1% and no lymphovascular invasion. Due to tumor invasion to the cecum and its large size (3–4 cm), the patient underwent right hemicolectomy. The final histopathology report of the resected specimen confirmed the diagnosis of NET of the appendix.

**Discussion:**

The clinical diagnosis of NETs of the appendix can be challenging due to their rarity and non-specific presentation. Symptoms of NETs of the appendix can mimic those of acute appendicitis, making it difficult to differentiate between the two conditions. Imaging studies, such as CT scans, can provide valuable information about the size and location of the tumor (Gomes et al. in World J Emerg Surg 10:60, 2015, Maggard et al. in Ann Surg 240:117–122, 2004, Burke et al. in Am J Surg Pathol. 9:661–674, 1985, Frilling et al. in Lancet Oncol 15:e8–e21, 2014). However, the definitive diagnosis is made through histopathological examination of the resected specimen. The treatment of NETs of the appendix depends on factors such as the size, location, and grade of the tumor. Small tumors confined to the appendix with no lymph-vascular invasion can be treated with appendectomy alone, while larger tumors or those that have spread beyond the appendix may require more extensive surgery, such as right hemicolectomy (Gomes et al. in World J Emerg Surg 10:60, 2015, Mestier et al. in Dig Liver Dis 52:899–911, 2020, Maggard et al. in Ann Surg 240:117–122, 2004, Burke et al. in Am J Surg Pathol. 9:661–674, 1985, Frilling et al. in Lancet Oncol 15:e8–e21, 2014, Pavel et al. in Neuroendocrinology 103:172–185, 2016). In some cases, additional treatments such as chemotherapy or radiation therapy may be recommended.

**Conclusion:**

This case report emphasizes the importance of considering NETs of the appendix in the differential diagnosis of acute appendicitis. Imaging studies can provide valuable information, but the definitive diagnosis is made through histopathological examination. The treatment approach for NETs of the appendix depends on various factors and requires a multidisciplinary approach for optimal management.

## Introduction

NETs of the appendix are uncommon and usually found incidentally during surgery for acute appendicitis or other unrelated conditions [1, 2]. They can range from asymptomatic incidental findings to clinically significant tumors with metastases [2, 3]. Here, we present a rare case of a NET of the appendix presenting as acute appendicitis.

## Case description

**Fig. 1:**
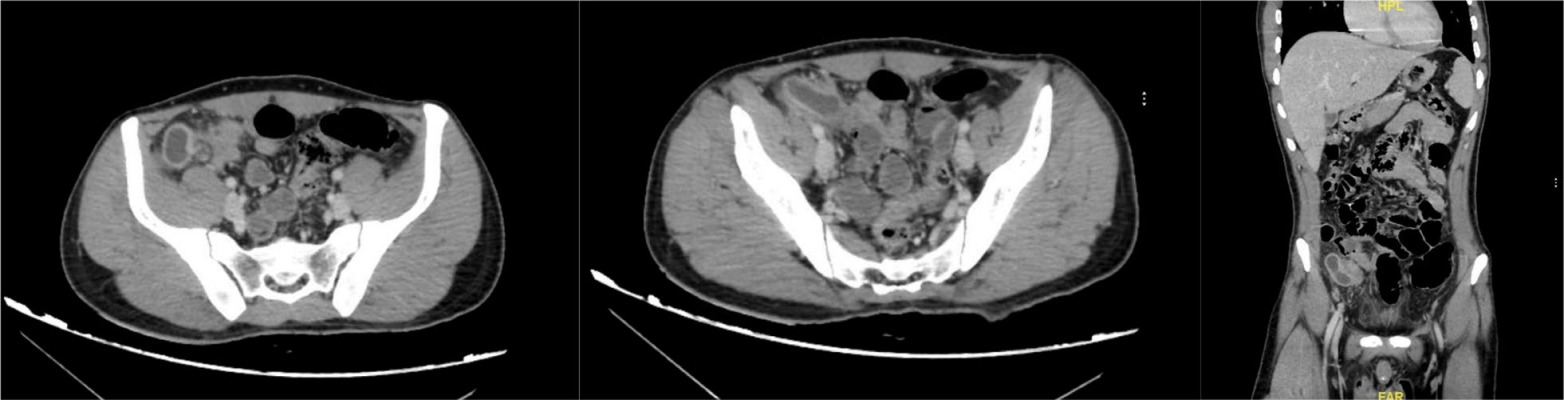
Axial, coronal CT appearance of the appendix

A 23-year-old male presented to the emergency department with a 2-day history of right lower quadrant abdominal pain, nausea, and vomiting. On examination, there was tenderness in the right lower quadrant with rebound tenderness, no guarding with normal vital signs. A CT scan showed a mucocoele of the appendix (Fig. 1). The patient underwent laparoscopic appendectomy, and the appendix was sent for histopathological examination. The final pathological report revealed a NET of the appendix with a Ki-67 index of 1% and no lymphovascular invasion, but invasion of the base of cecum with positive margins, size (3–4 cm) and grade.

**Fig. 2:**
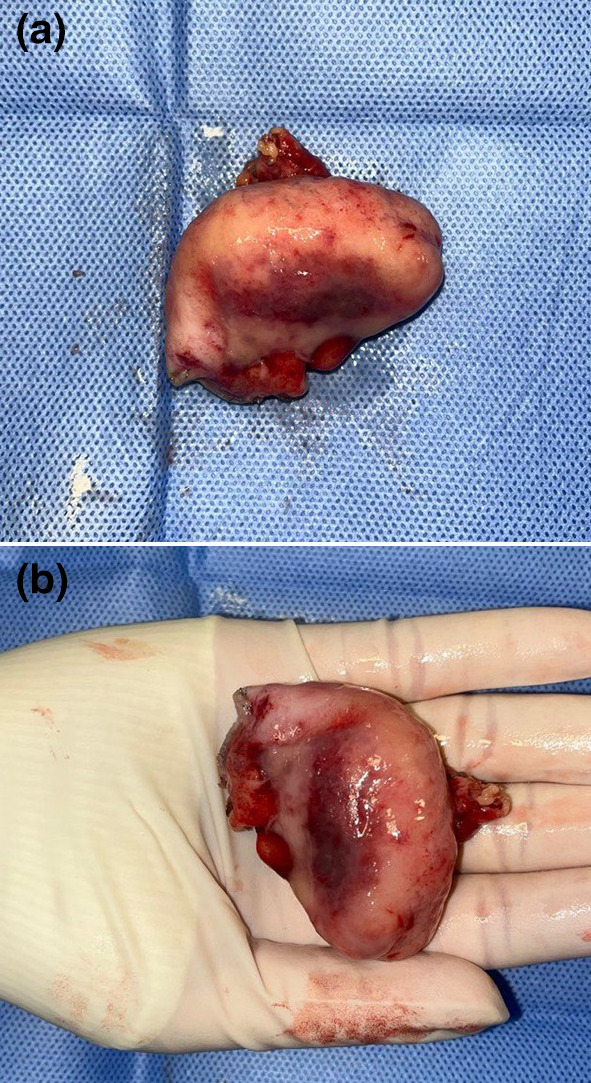
Gross appearance of the resected appendix

The patient was subsequently based on histopathological findings the patient was referred for right hemicolectomy. The final histopathology report of the resected specimen rt hemi confirmed the diagnosis of NET of the appendix (Fig. 2).

### Intra-operative approach and findings

**Fig. 3:**
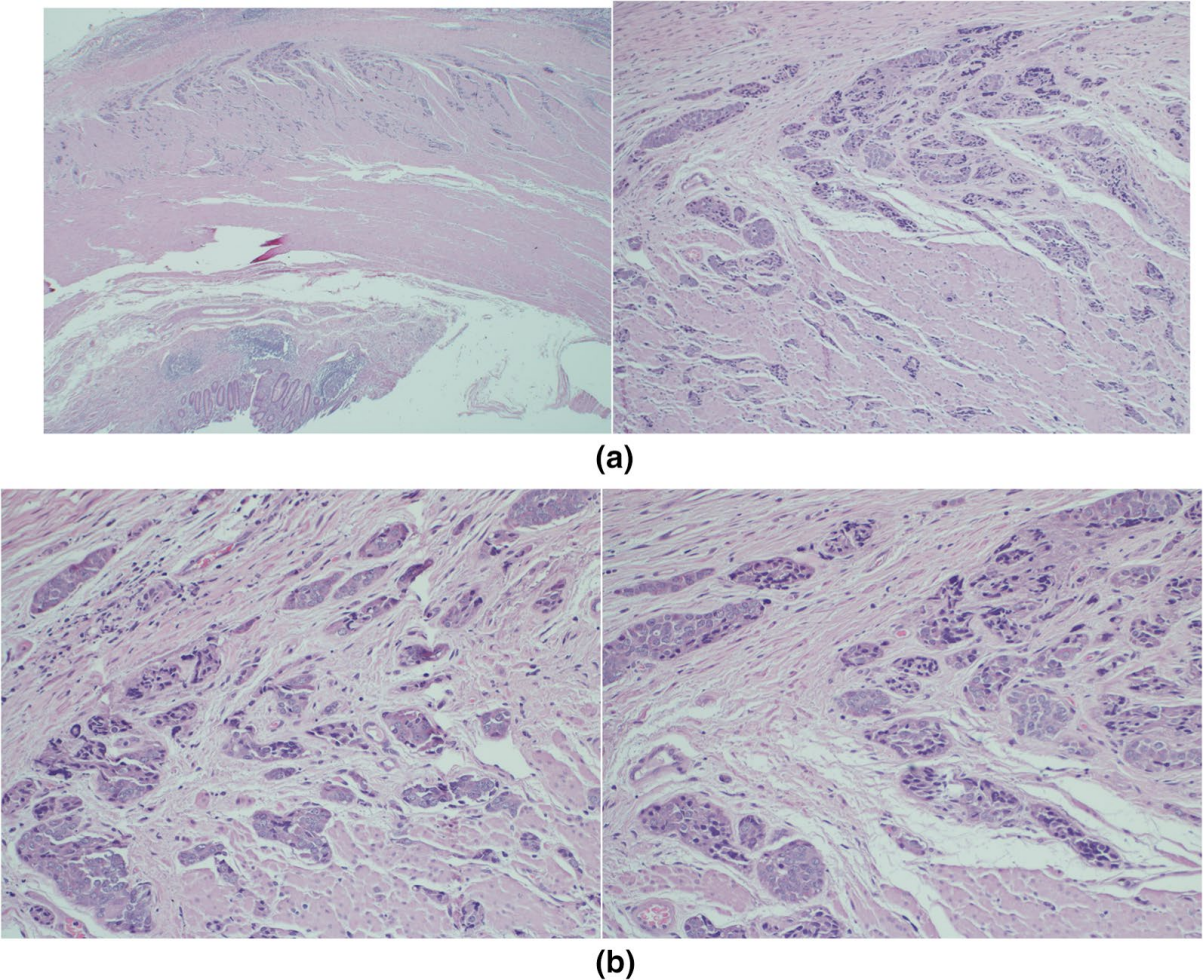
**A** This high-power view shows the tumor invading muscularis propria. **B** High magnification shows polygonal tumor cells arranged in nests and glandular growth pattern. Cells are monomorphic with round nuclei and finely stippled chromatin

**Fig. 4:**
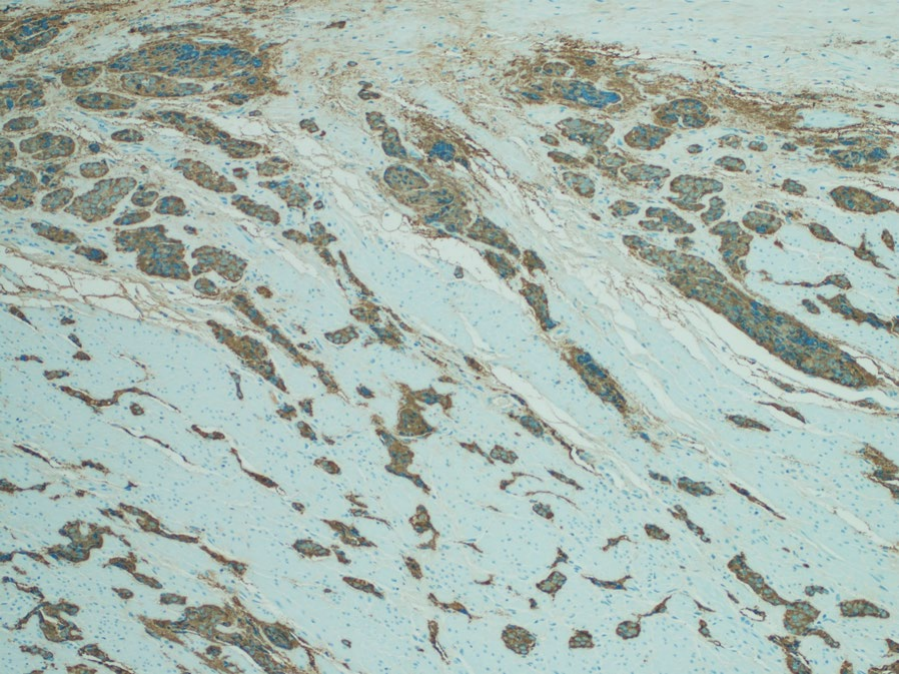
Shows diffusely strong positive staining for chromogranin

**Fig. 5:**
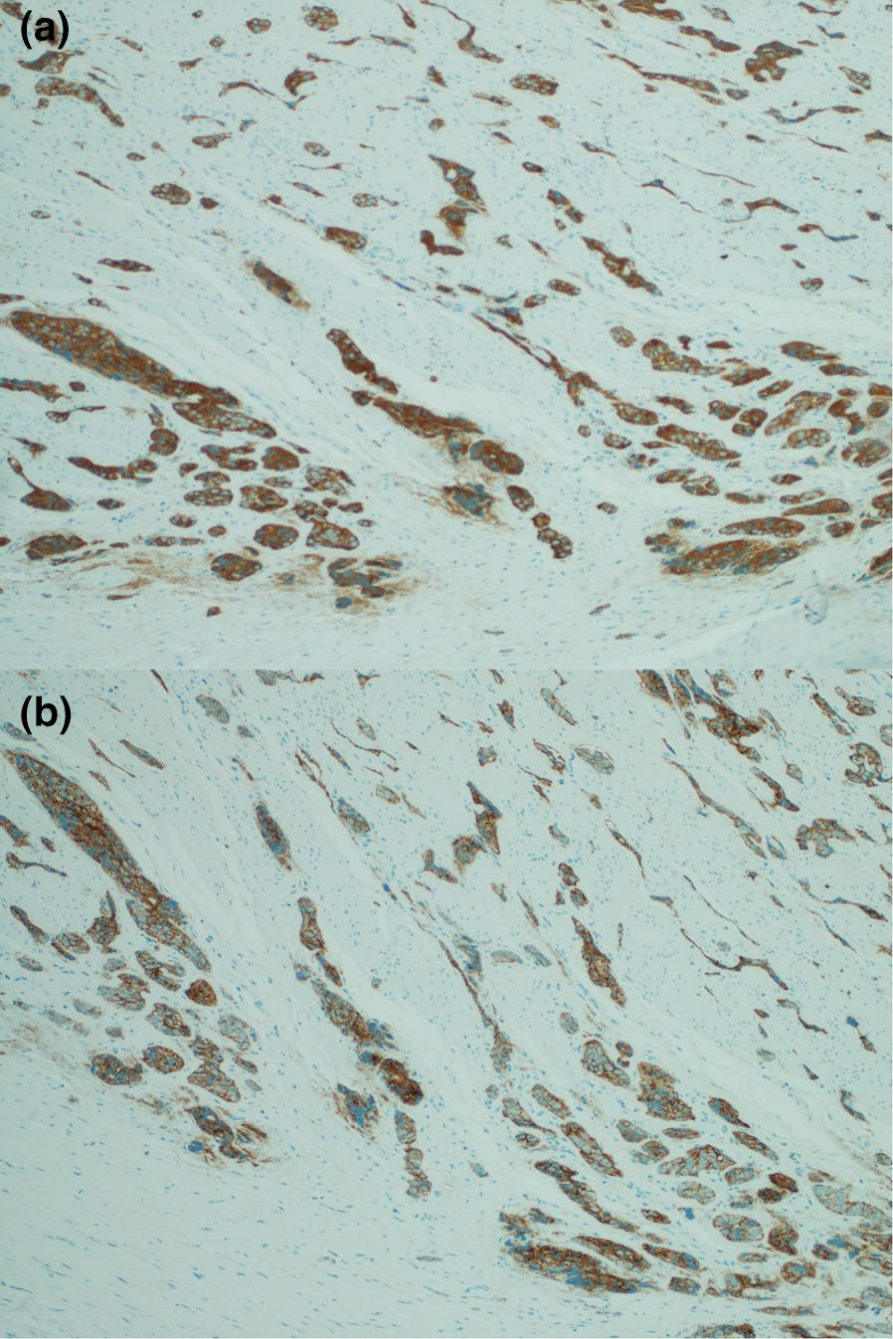
**a**, **b** Shows diffusely strong positive synaptophysin and diffusely strong positive CD56

A 12-mm trocar was placed through the supra-umbilical incision to approach the intraperitoneal cavity using the open Hasson technique. A pneumoperitoneum was made by the insufflation of carbon dioxide. The table was kept in Trendelenburg position with a 15° left tilt. A 30° telescope was introduced through the umbilical port for diagnostic laparoscopy, and complete abdominal examinations were done. Diagnostic laparoscopy revealed approximately 3–4 cm large mucocele of the appendix. Two 5- and 10-mm ports were placed in the left upper quadrant and the suprapubic area. The appendiceal artery was isolated after separating the mesoappendix with the help of bipolar cautery (ligasure). Following this, the base of the appendix was ligated at the ileocecal junction and divided by using an endoscopic stapling device (Multifire Endo GIA, 60 mm) which was used to perform the partial resection of cecum. The appendix was retrieved in a plastic bag through the umbilical port after careful minimal handling. Hemostasis was obtained. The umbilical port site wound was closed with j (needel). The patient tolerated the procedure, he started oral feeding 6 h post-operation and solid food on the next day. He was discharged on the third postoperative day without active complaint. Pathology showed the final report revealed a NET of the appendix with a Ki-67 index of 1% and no lymphovascular invasion, but invaded the base of cecum with positive margins (Figs. 3, 4, 5).

## Discussion

The diagnosis of NETs of the appendix is challenging due to the rarity of these tumors and their non-specific presentation. The symptoms of NETs of the appendix can mimic those of acute appendicitis, making it difficult to distinguish between the two conditions. Imaging studies such as CT scans can provide valuable information regarding the size and location of the tumor [4, 5].

Low-grade appendiceal mucinous neoplasm (LAMN) typically appears as a low-attenuation cystic lesion on CT scans, often accompanied by calcifications and wall thickening [4–6]. On the other hand, neuroendocrine tumors (NETs) of the appendix may present as solid masses with enhancement on contrast-enhanced CT scans [5, 6]. However, it is important to note that these imaging features are not always definitive for distinguishing between LAMN and NETs. Histopathological examination of the resected specimen remains crucial for accurate diagnosis [1, 2, 5, 6]. However, the definitive diagnosis of NET of the appendix is made by histopathological examination of the resected specimen [7, 8].

The treatment of NETs of the appendix depends on the size, location, and grade of the tumor. Small tumors that are confined to the appendix with no lymph-vascular invasion can be treated with appendectomy alone, whereas larger tumors or those that have spread beyond the appendix may require more extensive surgery, such as right hemicolectomy [9, 10]. In some cases, additional treatments such as chemotherapy or radiation therapy may also be recommended [11].

When the appendectomy margins are positive for tumor involvement, it is generally recommended to perform additional resection to achieve clear surgical margins. However, in this case, the decision for right hemicolectomy was made based on the invasion of the tumor into the cecum and the large size of the tumor (3–4 cm). The aim was to achieve complete resection and ensure adequate oncological clearance. The decision for right hemicolectomy was made in consultation with the multidisciplinary team, considering the extent of tumor involvement and the desire for optimal management [8, 12, 13].

Multidisciplinary treatment is important in cases where the size, location, and grade of the tumor warrant a comprehensive approach. Larger tumors or tumors with evidence of lymph node involvement, distant metastases, or invasion into adjacent structures often require a multidisciplinary team approach [7, 8, 12]. Collaboration between surgeons, oncologists, and radiologists can help determine the most appropriate treatment strategy, which may involve a combination of surgical resection, chemotherapy, and/or radiation therapy. Involving specialists in gastrointestinal oncology can provide valuable input in managing these complex cases [8, 14].

## Conclusion

This case report highlights the importance of considering NETs of the appendix in the differential diagnosis of acute appendicitis. Imaging studies such as CT scans can provide valuable information, but the definitive diagnosis of NET of the appendix is made by histopathological examination of the resected specimen. The treatment of NETs of the appendix depends on the size, location, and grade of the tumor. A multidisciplinary approach is essential for the optimal management of these rare tumors.

## Data Availability

The datasets used during the current study are available from the corresponding author upon request.

## Abbreviations

NETs: Neuroendocrine tumors
LAMN: Low-grade appendiceal mucinous neoplasm
